# The Oriental Plague; Its Cause, and Means of Preventing It

**Published:** 1838-01

**Authors:** 


### ART. XVI.

1. *Die Pest des Orients; wie sie entsteht und verhütet wird, drei Bücher.* Von Dr. C. J. LORINSER. *Berlin*, 1837. 8vo. pp. 461.

*The Oriental Plague; its Cause, and the Means of preventing it, in three Books.* By Dr. C. J. LORINSER.

2. *De Peste Antoniniana Commentatio. Scripsit* JUST. FRID. CAR. HECKER, M.D.—*Berolini*, 1835.

*An Essay on the Plague which prevailed in the Reign of Antoninus.* By Dr. J. F. C. HECKER, Professor of the History of Medicine in the University of Berlin, &c.—*Berlin*, 1835. 8vo. pp. 29.

ALTHOUGH works of the kind now before us afford no results of immediate practical application, they must be regarded as highly important in relation to our general knowledge of epidemic diseases. The investigation of the history of former or distant plagues may throw light upon those with which we are ourselves visited or threatened. The contemplation of such visitations on the great scale, and when the mind of the enquirer is freed from the many causes which disturb the judgment when he himself is a witness of the scene, is likely to lead to views and principles, both in regard to pathology and etiology, of a more enlarged and comprehensive character. Therefore it is that we are always gratified by the publication of treatises like the present, which, whatever their defects, bear the impress of honesty of purpose, industry, and learning. As the thick volume of Dr. Lorinser is valuable chiefly as a work of reference to persons engaged in similar enquiries, we shall merely indicate its general character and contents in this place. Of the matter contained in Dr. Hecker's classical little essay we shall give a brief outline and analysis, as it is at once interesting and in many respects novel, although conversant with times so remote.

I. It is with regret that Dr. Lorinser views the apathy with which the physicians of the present day regard the fearful scourge of the Plague, and it becomes one of his chief objects in the work before us to vindicate its claims to occupy a share of their attention commensurate with the deadliness of the malady. We do not perceive, however, that the author has enjoyed any peculiar advantages of studying this fatal disease; on the contrary, it appears that he has not had the opportunity of observing it personally; but he calculates upon the attention of his readers, chiefly, from the great importance which ought to be attached to the consideration of all measures which tend to limit its ravages and prevent its irruption into Europe, and because of his having enjoyed the opportunity of inspecting some of the numerous quarantine establishments, which are scattered over the Turco-Austrian and Turco-Russian frontiers.

Dr. Lorinser is evidently a man of considerable knowledge, and well conversant with all the standard works relating to the plague. His first book is devoted to a critical review of the preceding original writers on this disease, from Thucydides and the Greeks down to Dr. Patrick Russell. From the consideration of these works he seeks to demonstrate that Egypt has always been the birthplace of the plague, from which, as from a centre of infection, it has invaded the neighbouring countries.

In his second book he discusses the topographical character of Egypt, and takes occasion to express his conviction that the plague is at first an endemic disease, dependent upon the particular physical nature of the country, which acquires its contagious properties only at a later stage. This opinion accords with that of many of the French physicians who accompanied Napoleon's expedition, and likewise with that of the commission despatched to Egypt by the French government in 1828, for the express purpose of ascertaining its cause. It has been attempted by some to prove that the origin of the plague dates from the period at which the Egyptians ceased to embalm their dead, and that its cause is to be sought for in the putrid emanations arising from the bodies which have been disinterred by the waters of the Nile during the season of inundation. Dr. Lorinser is not satisfied as to this being an influential cause of the disease, but he believes that its periodic origin will be found to be owing to the unhealthy vapours which arise from the plains of the Delta after the return of the waters to their accustomed channel, whether they be contaminated by the corruption of animal or vegetable matter.

Two conditions are necessary for the propagation of the plague; the disease must in the first place be produced by the unhealthy emanations of the flooded plains, and secondly there must exist a peculiar state of the atmosphere favorable to its extention to countries too remote to suffer primarily from the poisonous vapours of the dank, reeking plains of Egypt. In the cities of Syria and Turkey it is not uncommon to observe the plague raging with violence for some weeks or months, and then gradually to die away, again to break forth with increased energy at some future period. But it does not follow that on each occasion a new focus of infection has been introduced from Egypt; it suffices that the seeds of infection have become latent, owing to the disappearance of that peculiar state of the atmosphere which is necessary to call into activity the principle of infection, and remained so till they are again vivified by some new atmospheric change.

In the third book we are brought to the consideration of those measures which have been put in practice by various European nations to limit the ravages of the disease. It contains a good account of the arrangements made by the consuls in the Levant to guard as much as possible against the danger of shipping infected goods, or of granting passports to suspected individuals; and also a long and very full description of the various quarantine establishments on the European shores of the Mediterranean, with a detail of the procedure of disinfection. We have besides a full account of the sanatory cordon established along the Turkish frontiers of Russia and Austria, with some interesting details as to the practical efficacy of the system.

The great want in Dr. Lorinser's work is the absence of all original ideas. It forms a good compilation, in many respects excellent, and evinces much labour and patience, but would be decidedly improved by the curtailment of about two-thirds of its pages. Its contents, in so far as they relate to quarantine establishments, are calculated more for the middle of last century than for the present day.

II. The leading principle which Dr. Hecker has followed out, as well in the present essay as in his larger works on the Black Death, the Dancing Mania, and the Sweating Sickness (so admirably translated by Dr. Babington,) is, that an epidemic disease is the natural result of the compound influences exerted by the customs, diet, and general mode of living of the people whom it invades, and not a sudden inexplicable outburst: so that Horace's rule, *nemo repentè fuit turpissimus,* applies to things as well as persons, and more years are required to arm an epidemic than to finish a scoundrel. Now, as every thing in this world is transitional, (the most opposite states being linked together by a thousand grades of approximation,) an unique condition of mankind has rarely, if ever, been witnessed; and hence diseases, whether epidemic or sporadic, which have been called unexampled, or extinct, are still to be detected, in a somewhat modified state, by the acute observer. Thus the dancing mania of the middle ages still survives in the Italian tarantula and even in St. Vitus's dance; sweating fevers are still seen in central Germany, particularly on the Maine, which have an evident resemblance to the English sweating sickness; and the *morbus cardiacus* of the ancients is nothing more than a scorbutic carditis, such as Dr. Crichton and Seidlitz have seen at Petersburg. (*See Brit. and For. Med. Rev.*, Vol. I. 259.)

Dr. Hecker is of opinion that the way was prepared for the pestilence which he describes, by extraordinary disturbances as well in the order of nature as in the frame of society. During the reign of Marcus Aurelius Antoninus and Lucius Verus, (the latter of whom reigned from A.D. 161 to 169, the former from 161 to 180,) tempests, earthquakes, inundations and scarcity of provisions combined in reducing whole nations to extreme misery. Vast clouds of locusts streaming from Asia, laid waste the fields of Europe: active warfare raged in the East, Illyria, Italy, and Gaul; and an increasing depravity of manners lent an additional shade to this gloomy picture.

*Origin of the Pestilence.* Preceded by these forerunners a plague broke out which must be numbered among the greatest in the annals of mankind, whether we consider its enormous mortality or the extent of the regions which it invaded. It is said to have first appeared at the city of Seleucia in Mesopotamia. The story runs that when Avidius Cassius had taken Seleucia, some of his soldiers rushed into the temple of Apollo Comeus, and searched a small aperture with the hope of finding something precious; on which the primitive infection burst forth from the secret hiding-places of the Chaldeans, and spread death and contagion from the confines of Persia to the Rhine and Gaul. Dr. Hecker thinks that the soldiers were infected by means of clothes, which perhaps were placed in the temple for that very purpose. The city was taken by Avidius Cassius A.D. 165.

*Progress of the Pestilence.* The disease had soon spread over the immense space from the Tigris to the Alps, as it broke out at Rome immediately after the triumph of the Emperors; the date of this ceremony, indeed, is placed by Eusebius as late as A.D. 168, but our author thinks it more probable that it took place in A.D. 166. It is certain that the pestilence delayed the war against the Marcomanni for three years; and in the year 168 it raged most violently at Rome, (but whether for the second or the third time is uncertain,) as we learn from the life of Galen. He was scared away from Rome by the plague, and retired into Campania; but not thinking himself safe even there, he sailed from Brundusium to Pergamus. About the end of the same year, or the beginning of the following one, (169,) he was recalled to Rome by the Emperors, and then set out for Aquileia; the pestilence having probably now subsided, so that greater military expeditions could be equipped. The assembling so many troops seems to have caused a recrudescence of the epidemic, yet not such as to have put a stop to the campaign, or obstructed the ordinary course of affairs. It had not entirely ceased, however, when Galen wrote his fifth book *de medendi methodo*, as appears from his expression "ὃν εἴη ποτὲ παύσεσθαι,"—would that at length it might cease! Now Galen wrote, as the learned agree, in his old age, and after A.D. 180. It was in this year that Marcus Aurelius was carried off, and, as the words used by Julius Capitolinus would lead one to suppose, by the pestilence; "on the seventh day he was much depressed, and would see only his son, whom he dismissed immediately, lest he should be infected; (septimo die gravatus est, et solum filium admisit, quem statim dimisit, ne in eum morbus transiret.)"

*Nature of the Disease.* The first symptom mentioned is a pustular eruption terminating in sores and crusts; sometimes, however, it was papular, and ended in desquamation; in the former case the cutis vera was affected, in the latter the epidermis alone. Galen calls it *ἐξανθήμα μέλανα, a black eruption;* the blackness showing that the disease was of a malignant and putrid type. The eruption was critical, those patients who had none, or only an imperfect form, being in the greatest danger. Our author declares this pestilent exanthema not to have been the small-pox, as that disease did not appear in Europe till the sixth century; a fact for the proofs of which he refers to an essay of his, entitled *die Pest im sechsten Jahrhundert* (the plague in the sixth century,) in the *Annalen der gesammten Heilkunde,* vol. x. p. 1.

Another symptom was a violent cough, with hoarseness, and fetid breath. One patient, a young man, with a bad cough and ulceration of the trachea, brought up a *crust*, i. e. a shred of lymph. Thucydides too, who described a pestilence of the same kind, mentions that the patients after sneezing were attacked with hoarseness and violent coughing. Galen's epidemic was likewise attended with a pestilential redness of the tongue, mouth, and fauces; and a fatal diarrhœa. On the whole, the disease seems to have possessed some features in common with both the small-pox and malignant scarlatina as observed in recent times; as in these, the mucous membranes of the bronchi and bowels participated with the skin in the morbid affection. Dr. Hecker however considers it as a peculiar disease, a true specimen of the plague of the ancients, of which he promises a full history at a future time.

---

